# Decreased Glucagon-Like Peptide-1 Is Associated With Calcific Aortic Valve Disease: GLP-1 Suppresses the Calcification of Aortic Valve Interstitial Cells

**DOI:** 10.3389/fcvm.2021.709741

**Published:** 2021-08-26

**Authors:** Fan Xiao, Qing Zha, Qianru Zhang, Qihong Wu, Zhongli Chen, Ying Yang, Ke Yang, Yan Liu

**Affiliations:** ^1^Department of Cardiology, Shanghai Ninth People's Hospital, Shanghai Jiaotong University School of Medicine, Shanghai, China; ^2^Department of Vascular and Cardiology, Ruijin Hospital, Shanghai Jiaotong University School of Medicine, Shanghai, China; ^3^Department of Endocrinology, The Second People's Hospital of Yunnan Province, Kunming, China

**Keywords:** glucagon-like peptide-1, calcific aortic valve disease, calcification, aortic valve interstitial cells, age

## Abstract

**Objectives:** This study explores the concentration and role of glucagon-like peptide-1 (GLP-1) in calcific aortic valve disease (CAVD).

**Background:** Calcific aortic valve disease is a chronic disease presenting with aortic valve degeneration and mineralization. We hypothesized that the level of GLP-1 is associated with CAVD and that it participates in the calcification of aortic valve interstitial cells (AVICs).

**Methods:** We compared the concentration of GLP-1 between 11 calcific and 12 normal aortic valve tissues by immunohistochemical (IHC) analysis. ELISA was used to measure GLP-1 in serum of the Control (*n* = 197) and CAVD groups (*n* = 200). The effect of GLP-1 on the calcification of AVICs and the regulation of calcific gene expression were also characterized.

**Results:** The GLP-1 concentration in the calcific aortic valves was 39% less than that in the control non-calcified aortic valves. Its concentration in serum was 19.3% lower in CAVD patients. Multivariable regression analysis demonstrated that GLP-1 level was independently associated with CAVD risk. *In vitro*, GLP-1 antagonized AVIC calcification in a dose- and time-dependent manner and it down-regulated RUNX2, MSX2, BMP2, and BMP4 expression but up-regulated SOX9 expression.

**Conclusions:** A reduction in GLP-1 was associated with CAVD, and GLP-1 participated in the mineralization of AVICs by regulating specific calcific genes. GLP-1 warrants consideration as a novel treatment target for CAVD.

## Introduction

Glucagon-like peptide-1 (GLP-1), a hormone of 30 amino acids, is derived from proglucagon (1). It can increase insulin's sensitivity in regulating blood glucose (2), which is an effect that partially reverses aging-related degenerative disease (3). Calcific aortic valve disease (CAVD) is a common and chronic heart valve disease (4) that exhibits aortic valve thickening and calcification (5). Calcific aortic valve disease involves chronic inflammatory responses (6), lipid accumulation (7, 8), extracellular matrix rebuilding (9, 10), and osteogenic-related gene activation (11, 12). Glucagon-like peptide-1 has shown benefits on cardiovascular function in both preclinical and clinical studies (13–15). Furthermore, a 5-week infusion of GLP-1 (2.5 pmol/kg/min) added to standard therapy in patients with heart function of New York Heart Association class III/IV improved the LV ejection fraction in both diabetic and non-diabetic patients (16). However, the relationship between GLP-1 and CAVD has not been elucidated.

Valve interstitial cells (VICs) are heterogeneous cells that present various phenotypes (myofibroblasts, fibroblasts, and smooth muscle-like cells) and they participate in the physiological function of the aortic valve (17). For instance, the risk factors for CAVD evoke osteogenic signaling, which turn innate quiescent valve interstitial cells (qVICs) into activated valve interstitial cells (aVICs) and facilitate them to differentiate into osteoblastic valve interstitial cells (obVICs) (4, 18, 19). Glucagon-like peptide-1 suppresses vascular smooth muscle cell (VSMC) mineralization by reducing osteogenic gene expression and activating arterial calcification (20). The phenotypic changes in VICs are the main cytological events leading to aortic valve calcification; however, whether GLP-1 regulates the phenotype of VICs is unknown.

In this work, we hypothesized that GLP-1 is differentially regulated in the serum and tissue of CAVD patients and that its concentration is associated with aortic valve calcification.

## Materials and Methods

### Patients

**Figure 1:**
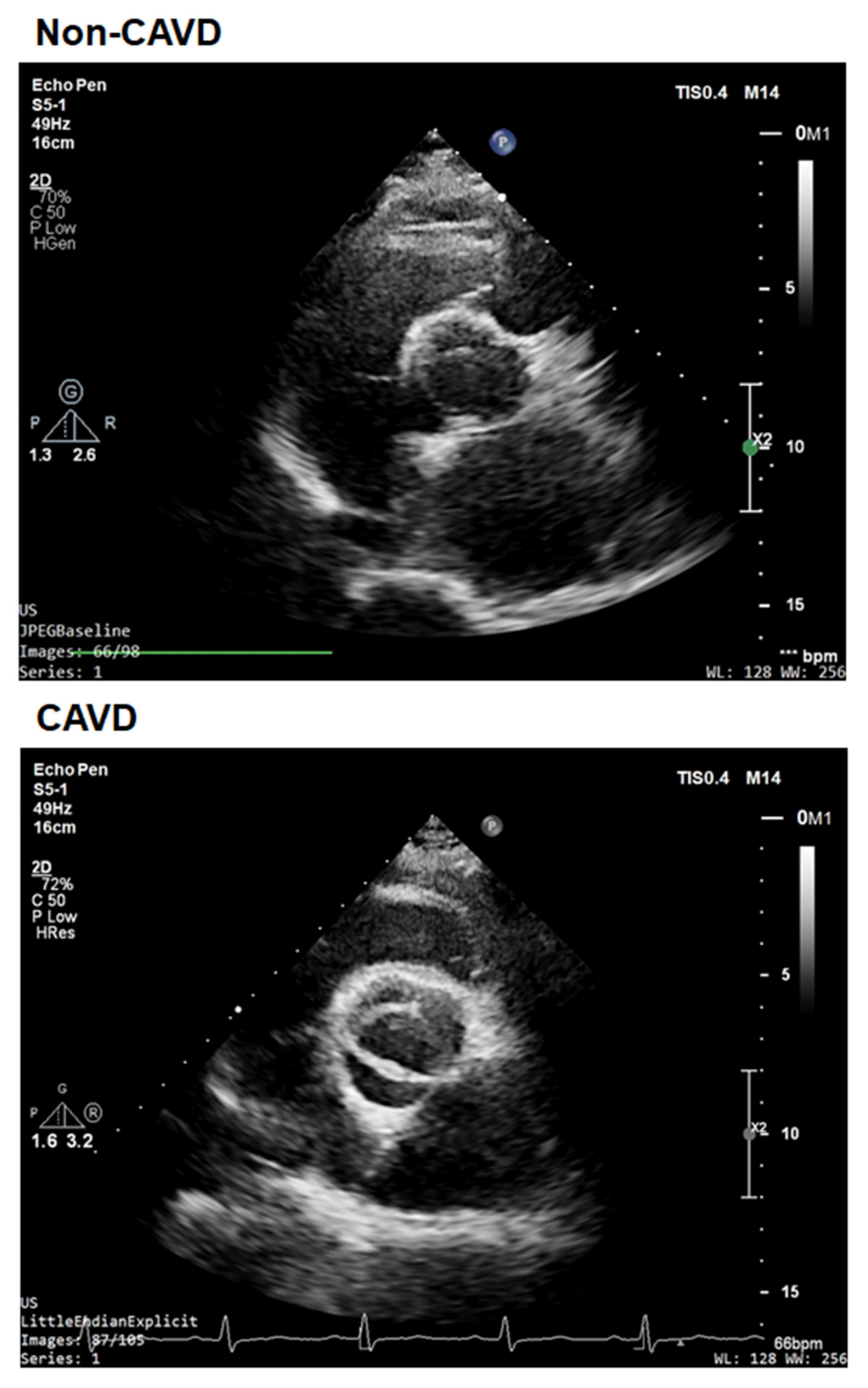
A representative echo image of non-CAVD (upper) and CAVD (bottom).

We performed a retrospective study on 200 aortic valves with calcific degeneration (CAVD) and 197 without calcific degeneration between January 2013 and August 2014 in patients recruited from the database of Shanghai Rui Jin Hospital who underwent echocardiographic screening. According to recommendations of the American Society of Echocardiography during hospitalization and patients entered into the screening procedure (21), and underwent standard transthoracic echocardiography and Doppler flow imaging. Calcific aortic valve disease was defined as opaque leaflets with focal areas of mild thickening and increased stiffness with or without an elevated peak trans-aortic valve flow velocity (≥2.0 m/s) (22) (Figure 1). Patients with a history of rheumatic disease, endocarditis, or an inflammatory disease were excluded. Detailed medical and family histories were recorded, and fasting blood samples were collected during physical check-up. The diagnosis of type 2 diabetes, hypertension, and coronary artery diseases (CAD)was made according to corresponding criteria of the American Diabetes Association (23), hypertension (24), and CAD (25) guidelines.

Human aortic valves with calcification were obtained from 11 patients who underwent valve replacement. Aortic valve leaflets were collected from the explanted hearts of 12 patients undergoing heart transplantation as normal aortic valves. The study protocol was approved by the Ethics Committee of Ruijin Hospital, Shanghai Jiaotong University School of Medicine, and written informed consent was obtained from all patients.

### Reagents and Antibodies

A High Sensitivity GLP-1 Active ELISA Kit (Cat# EZGLPHS-35K, Millipore, MA, USA) was used to measure GLP-1 in serum. Recombinant GLP-1 peptide (Human, Cat #SCP0153), a 3,3′-Diaminobenzidine Liquid Substrate System (Cat# D3939), an Alkaline Phosphatase Diethanolamine (ALP) Activity Kit (Cat# AP0100), Alizarin Red S (Cat# A5533), and a Masson Stain Kit (Cat# HT15) were purchased from Sigma-Aldrich (MO, USA). Primary antibodies were used to detect GLP-1 (Cat# ab22625, Abcam, MA, USA), RUNX2 (Cat# 12556, Cell Signaling Technology, MA, USA), MSX2 (Cat# ab69058, Abcam, MA, USA), SOX9 (Cat# 82630, Cell Signaling Technology, MA, USA), BMP2 (Cat# ab14933, Abcam, MA, USA), BMP4 (Cat# ab39973Abcam, MA, USA), and β-actin (Cat# 4970, Cell Signaling Technology, MA, USA) in immunohistochemical (IHC) or immunoblot assays. The secondary antibodies were horseradish peroxidase (HRP)-conjugated anti-rabbit antibodies (Cat# 7074, Cell Signaling Technology, MA, USA) or Alexa Fluor 594- or Alexa Fluor 488-conjugated anti-rabbit antibodies (Cat# R37119 or Cat# A27034, Thermo Fisher Scientific, NY, USA). Fetal bovine serum (FBS, Cat# 16000044), DMEM:F12 culture medium, penicillin, and streptomycin were from Gibco BRL (NY, USA).

### Primary Aortic Valve Interstitial Cell Culture

Human aortic valve leaflets from the explanted hearts were gathered to culture the primary aortic valve interstitial cells (AVICs) (26). Briefly, valve leaflets were subjected to collagenase digestion and gently scraped to expose the endothelial layer. The leaflets were then cut into microscopic pieces (1–2 mm^2^) and cultured in DMEM:F12 (1:1) supplemented with 20% FBS, L-glutamine (2 mmol/L), penicillin (100 U/ml), and streptomycin (100 μg/ml). Cells were grown with 5% CO_2_ at 37°C. Upon reaching 80% confluency, the AVICs were passaged using trypsin-EDTA. AVICs between passage 3 and 8 were used for experiments.

### Immunohistochemistry

Human calcific (*n* = 11) and non-calcific aortic valves (*n* = 12) were used for histological and immunochemical analysis. Samples were fixed in 4% paraformaldehyde overnight and cut into serial cryosections (5 μm thickness). Sections were used for hematoxylin and eosin (H&E) staining, Alizarin Red S staining, and Masson trichrome staining. Glucagon-like peptide-1 in the valves was detected by IHC using anti-GLP-1 antibody (1:50). After incubation with HRP-conjugated secondary antibodies (1:100), sections were incubated with 3,3′-diaminobenzidine.

The sections of primary AVICs were used for immunofluorescence analysis. The cells were immune-stained with anti-RUNX2 (1:50), anti-MSX2 (1:50), anti-SOX9 (1:50), anti-BMP2 (1:50), or anti-BMP4 (1:50) for 12 h at 4°C and incubated with Alexa Fluor 549- or Alexa Fluor 488-conjugated secondary antibody (1:1,000).

### *In vitro* Calcification of AVICs

Primary AVICs were isolated from the human aortic valve. Aortic valve interstitial cell calcification was induced in osteogenic medium containing DMEM and supplemented with 15% FBS, 50 mg/ml ascorbate-2-phosphate, 10 nM dexamethasone, and 10 mM β-glycerol phosphate (27). The culture medium was changed every 48–72 h, and the cells were harvested for 3 weeks. Aortic valve interstitial cell calcification was determined by Alizarin Red S staining. The cells were washed in distilled water and then exposed to freshly prepared 2% Alizarin Red S (pH to 4.1–4.3) for 5 min (red/orange as positive staining). For quantitative analysis of Alizarin Red S staining, the dye was released from the cell matrix by incubating with cetylpyridinium chloride for 15 min. The released dye was quantified by spectrophotometry at 540 nm. Alkaline Phosphatase Diethanolamine activity was determined using the spectrophotometric measurement of the p-nitrophenol level in the AVICs (28). The amount of Alizarin Red S staining and ALP activity were normalized to the total amount of cellular protein.

### Quantitative Real-Time PCR

Total RNA was extracted as described above. Briefly, 5 μg of total RNA was reverse-transcribed into cDNA using a reverse transcription system (Promega, WI, USA). PCR amplification was performed with Power SYBR Green PCR Master Mix (Applied Biosystems, CA, USA) in a StepOne system (Applied Biosystems). The oligonucleotides used in quantitative real-time PCR analysis are listed in Supplementary Table 1. The gene expression levels were normalized to beta-actin, and the data were analyzed with StepOne software v2.1 (Applied Biosystems).

### Western Blot

Cells were lysed with the ProteoJET Mammalian Cell Lysis Reagent (Fermentas, MD, USA) to extract cytoplasmic proteins. Equal amounts of protein extracts were subjected to 10% SDS/PAGE and blotted onto a poly (vinylidene difluoride) membrane. The membrane was blocked and probed overnight at 4°C with antibodies against RUNX2 (1:1,000), MSX2 (1:1,000), SOX9 (1:100), BMP2 (1:500), BMP4 (1:500), and β-actin (1:2,000), followed by incubation with HRP-conjugated secondary antibodies (1:5,000) for 1 h at room temperature. Blots were developed using an ECL detection system (Millipore, MA, USA). Each image was captured and the intensity of each band was analyzed with Quantity One (Bio-Rad).

### Statistical Analysis

We performed statistical analyses with SPSS software (version 20). All tests were two-tailed, and a *p*-value of < 0.05 was regarded as statistically significant. Demographic and clinical characteristics were compared between the CAVD and non-CAVD groups. Fisher's exact test was employed for categorical variables, and a two-independent-samples *t*-test or Wilcoxon rank-sum test was employed for numerical variables to evaluate between-group differences. To analyze risk factors for CAVD, candidate variables were decided *a priori* by referral to previous reports. Using these variables, we performed multivariable logistic regression analyses with a backward stepwise selection procedure. Odds ratios (ORs) and 95% CIs were calculated. For ALP and ALZ, repeated-measures ANOVA with the group as a fixed factor was employed to compare the differences between two groups.

## Results

### Distribution of GLP-1 in Calcific Aortic Valve

**Figure 2:**
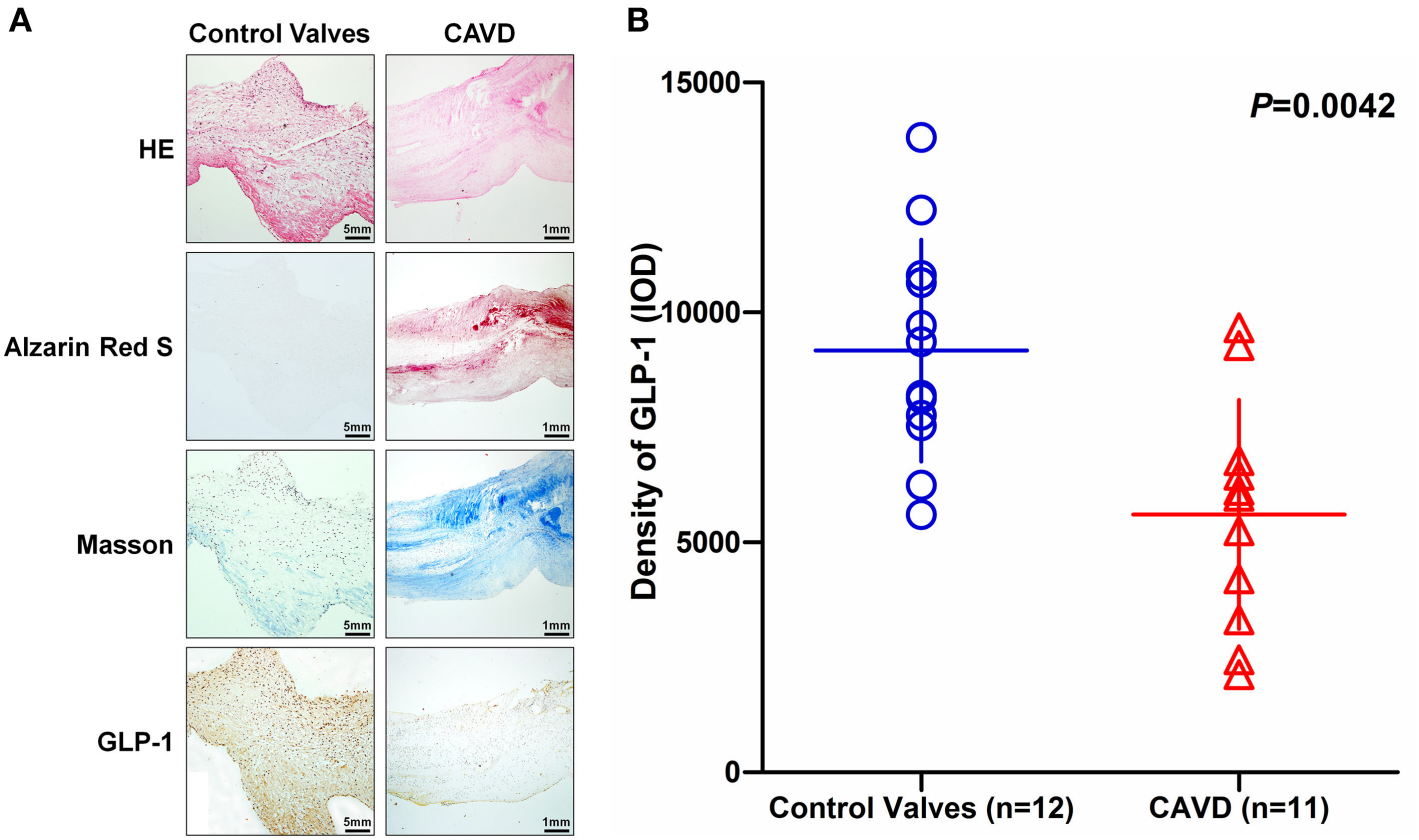
The distribution of GLP-1 in aortic valves with or without calcification. Human aortic valves with calcification (*n* = 11) that underwent valve replacement operation and without calcification (*n* = 12) undergoing heart transplantation were assessed by histological and immunochemical analysis. **(A)** Sections were stained with hematoxylin and eosin, Alizarin Red S, and Masson trichrome staining. IHC stains of GLP-1 and counterstained with hematoxylin. The results of the Non-CAVD valves (left line) are shown at 100 × magnification, and the results of CAVD (right line) are shown at 20 × magnification. **(B)** The concentration of GLP-1 detected by IHC was determined by assessing its staining with Image-Pro Plus 6.0. The results are shown as the integrated optical density (IOD)/area. The Non-CAVD (*n* = 12) and CAVD valves (*n* = 11) are representative of three independent experiments, and five different fields in each section were detected (the *P*-value was control valves compared with CAVD).

Based on the histological analysis, the calcific aortic valve exhibited structural thickening, mineralization and ECM remodeling (Figure 2A) and the demographic and clinical characteristics of the study participants were summarized in Table 1. The IHC analysis showed that GLP-1 was mainly evenly distributed in the rich region of VICs of control aortic valves, but GLP-1 was prominently distributed in the non-mineralized areas of calcific aortic valves (Figure 2A). The integrated optical density (IOD) of the GLP-1 level was calculated in 12 control valves and 11 calcific aortic valves. Compared with the control valves, the concentration of GLP-1 decreased in the calcific aortic valves by 39% (CAVD: 5,606 ± 750.4; vs. Control valves: 9,170 ± 695.9; *P* = 0.0042) (Figures 2A,B).

**Table 1:** Baseline characteristics of aortic valves obtained from patients.

**Variable**	**Non-CAVD (*n* = 12)**	**CAVD (*n* = 11)**	***p***
Age, years	62 (58–66)	72 (65–77)	<0.01
BMI, kg/m^2^	26 (24–27)	23 (21–25)	0.02
Male, *n* (%)	10 (83)	10 (91)	NS
Smoking status, *n* (%)	2 (17)	3 (27)	NS
Alcohol consumption, *n* (%)	1 (8)	3 (27)	NS
Hypertension, *n* (%)	8 (67)	7 (64)	NS
CHD, *n* (%)	12 (100)	11 (100)	NS
DM, *n* (%)	3 (25)	0 (0)	NS
SBP, mmHg	141 (129–156)	131 (115–143)	NS
DBP, mmHg	80 (71–88)	72 (58–78)	NS
Fasting glucose, mmol/L	4.96 (4.12–5.94)	5.40 (4.88–5.88)	NS
HbA1c, %	6.5 (5.8–7.0)	5.7 (5.5–6.0)	0.03
TG, mmol/L	1.77 (1.06–2.48)	1.35 (0.69–1.96)	NS
TC, mmol/L	4.07 (3.03–4.81)	4.59 (3.37–5.55)	NS
HDL-c, mmol/L	0.88 (0.67–1.06)	1.08 (0.94–1.26)	0.04
LDL-C, mmol/L	2.55 (1.60–3.44)	2.78 (1.77–3.17)	NS
Lp(a), g/L	0.21 (0.07–0.30)	0.30 (0.10–0.31)	NS
BUN, mmol/L	5.17 (4.33–5.80)	6.55 (4.20–8.20)	NS
SCr, μmol/L	83.08 (78–91)	104.55 (76.00–123.00)	NS
eGFR, ml/min	84.18 (81.25–89.88)	67.89 (52.70–88.60)	NS
Cystatin, mg/L	1.10 (0.89–1.20)	1.39 (1.14–1.72)	0.04
Ca, mmol/L	2.22 (2.13–2.32)	2.18 (2.07–2.31)	NS
P, mmol/L	1.17 (1.09–1.29)	0.99 (0.85–1.14)	0.02
Aortic valve area, cm^2^	–	0.74 ± 0.07	–
Aortic mean gradient, mmHg	–	47 ± 3	–
Glucose lowering therapy, *n* (%)	3 (25)	0 (0)	NS
Statins, *n* (%)	11 (92)	11 (100)	NS

*Continuous variables are presented as the median (25th−75th percentile), and categorical variables are expressed as n (%). For continuous variables, Mann-Whitney U-tests were performed to assess differences. Differences in proportions were analyzed by 2 × 2 chi-square tests*.

### GLP-1 Regulates Calcification of AVICs

**Figure 3:**
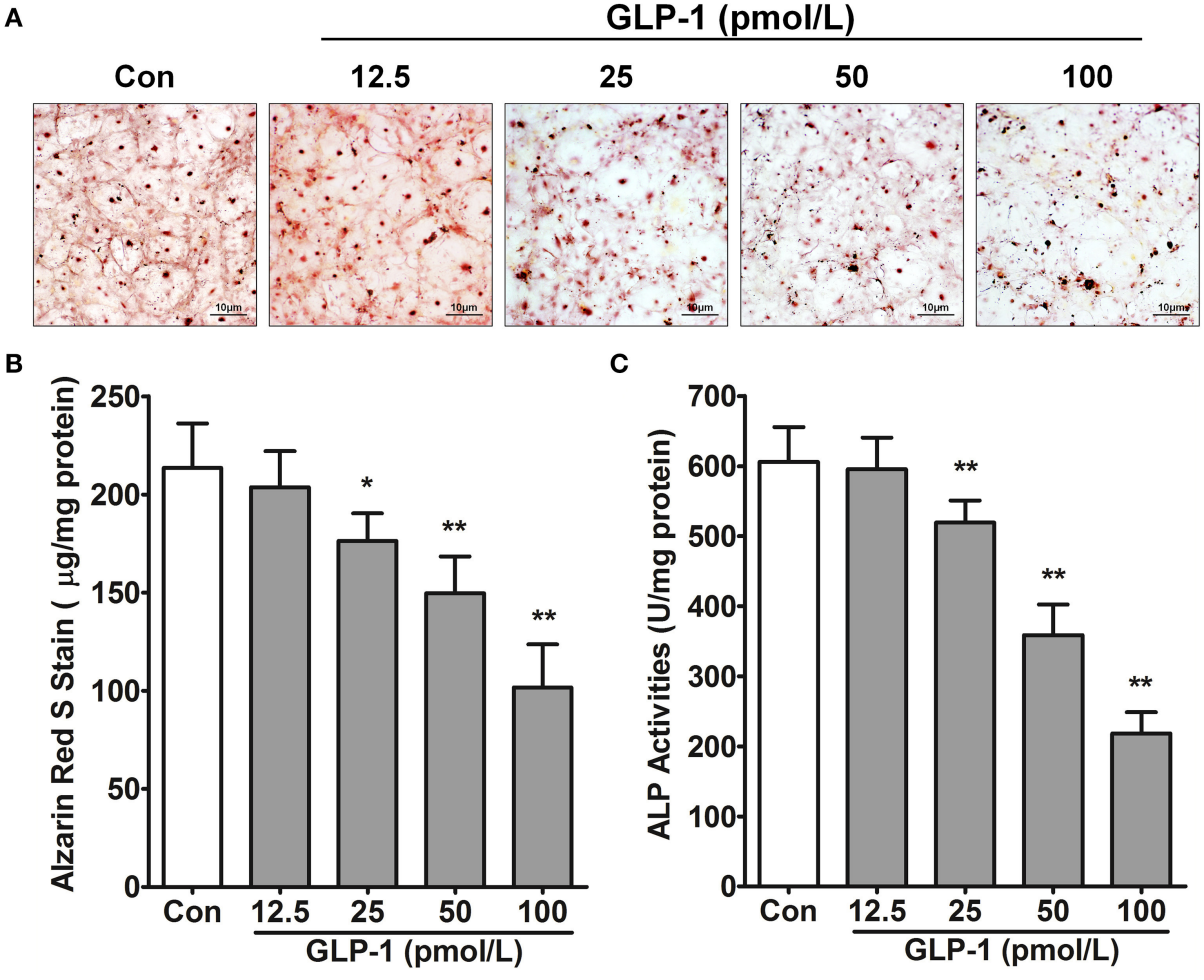
GLP-1 inhibits AVIC calcification. In osteogenic medium, the primary AVICs were incubated with increasing doses (12.5, 25, 50, and 100 pmol/L for 21 days) of GLP-1. The group without the GLP-1 treatment was used as a control (Con). **(A)** Alizarin Red S was used to stain the calcification cells. The pictures were taken using Image-Pro Plus 6.0. Alizarin Red S staining is shown at 400 × magnification. **(B)** Quantification of Alizarin Red S staining. **(C)** Quantification of ALP activation in AVICs. The dye was extracted and quantified as described in the Methods section (*n* = 6, mean ± SD, *P < 0.05, **P < 0.01 compared with Con).

Based on the GLP-1 concentration of patients' serum in this study (Table 2), we treated AVICs with different doses of GLP-1 to identify the effect of GLP-1 on calcification *in vitro*. Alizarin Red S staining showed that the osteogenic medium (used in the Controls) induced AVIC mineralization, but a higher dose (25–100 pmol/L) of GLP-1 reversed the calcification of AVICs (Figure 3A). The Alizarin Red S dilution results [Control = 213.5 ± 9.248 μg/mg; GLP-1 (12.5 pmol/L) = 203.7 ± 7.535 μg/mg vs. Control, *P* = 0.318; GLP-1 (25 pmol/L) = 176.3 ± 5.754 μg/mg vs. Control, *P* = 0.033; GLP-1 (50 pmol/L) = 149.7 ± 7.632 μg/mg vs. Control, *P* = 0.007; GLP-1 (100 pmol/L) = 101.7 ± 8.950 μg/mg vs. Control, *P* = 0.001] and the activation of ALP [Control=605.5 ± 20.53 U/mg; GLP-1 (12.5 pmol/L) = 595.7 ± 18.26 U/mg vs. Control, *P* = 0.513; GLP-1 (25 pmol/L) = 519.5 ± 12.73 U/mg vs. Control, *P* = 0.002; GLP-1 (50 pmol/L) = 358.7 ± 17.94 U/mg vs. Control, *P* = 0.001; GLP-1 (100 pmol/L) = 218.3 ± 12.41 U/mg vs. Control, *P* < 0.001] also showed that GLP-1 reduced AVIC calcification in a dose-dependent manner (Figures 3B,C).

**Table 2:** Clinical characteristics of the control and CAVD groups.

	**Control (*n* = 197)**	**CAVD (*n* = 200)**	***P***
Age, yrs	59.02 ± 8.59	74.14 ± 7.67	<0.001
Male, *n* (%)	111 (56.3)	123 (61.5)	NS
Body mass index, kg/m^2^	24.65 ± 3.39	25.13 ± 3.46	NS
Active smokers, *n* (%)	59 (29.9)	48 (24.0)	NS
Alcohol, *n* (%)	27 (13.7)	18 (9.0)	NS
Hypertension, *n* (%)	127 (64.5)	154 (77.0)	0.008
Diabetes, *n* (%)	63 (32.0)	61 (30.5)	NS
Coronary heart disease, *n* (%)	134 (68.0)	177 (88.5)	<0.001
Systolic blood pressure, mmHg	77.07 ± 11.70	74.42 ± 11.43	0.023
Diastolic blood pressure, mmHg	131.49 ± 17.73	139.78 ± 20.68	<0.001
Fasting glucose, mmol/L	5.48 ± 1.86	5.52 ± 2.21	NS
HbA1c, %	6.21 ± 1.167	6.38 ± 1.23	NS
Triglycerides, mmol/L	1.78 ± 1.13	1.56 ± 0.86	0.025
Total cholesterol, mmol/L	4.08 ± 1.06	3.91 ± 1.19	NS
LDL, mmol/L	1.13 ± 0.26	1.07 ± 0.29	0.044
HDL, mmol/L	2.39 ± 0.88	2.32 ± 0.98	NS
Lipoprotein(a), g/L	0.12 (0.20)	0.14 (0.30)	0.026
γ-Glutamyl transpeptidase, U/L	19.00 (20.00)	21.00 (14.00)	NS
Blood urea nitrogen, mmol/L	5.06 ± 1.29	6.03 ± 2.40	<0.001
Creatinine, mmol/L	74.07 ± 16.07	86.38 ± 24.25	<0.001
eGFR(CKD-EPI), mL/min	90.39 ± 17.62	72.29 ± 18.23	<0.001
Peak aortic transvalvular velocity, m/s	2.10 ± 0.21	2.14 ± 0.22	NS
Systolic pulmonary artery pressure, mmHg	34.81 ± 2.25	35.21 ± 1.98	NS
Aortic valve mean gradient, mmHg	4.51 ± 1.01	4.72 ± 1.12	NS
Metformin, *n* (%)	35 (17.8)	28 (14.0)	NS
Statin, *n* (%)	163 (82.7)	167 (83.5)	NS
GLP-1, pmol/L	14.00 (9.68)	11.29 (6.75)	<0.001

*Values are expressed as the mean ± SD, number (%), or median (interquartile range)*.

*CAVD, calcific aortic valve disease; HDL, high-density lipoprotein; LDL, low-density lipoprotein; NS, not significant*.

**Figure 4:**
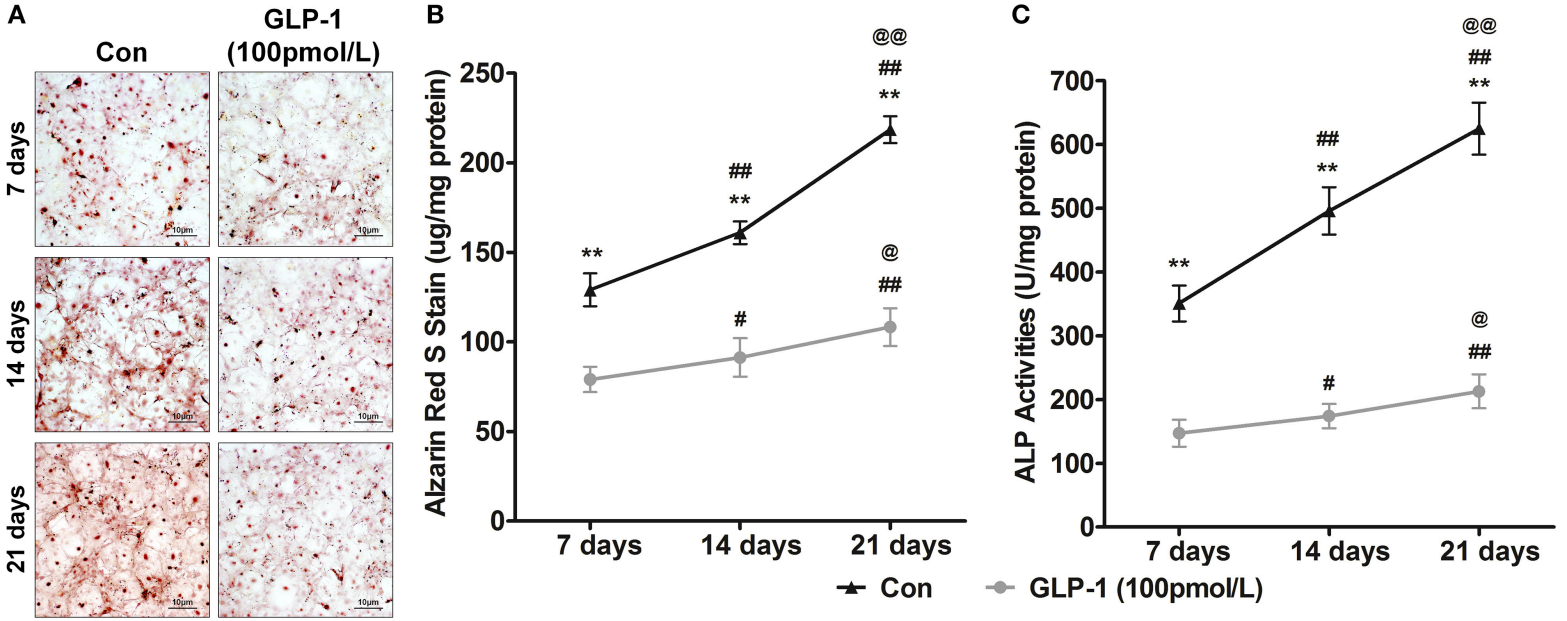
GLP-1 postpones the age-induced mineralization of AVICs. To determine the effect of GLP-1 on age-induced mineralization in AVICs, primary AVICs were cultured for 7, 14, and 21 days in osteogenic medium plus GLP-1 (100 pmol/L) or not. The group without the GLP-1 treatment was used as a control (Con). **(A)** Alizarin Red S stained the calcification cells, and the pictures were taken using Image-Pro Plus 6.0. Alizarin Red S staining is shown at 400 × magnification. **(B)** Quantification of Alizarin Red S staining. **(C)** Quantification of ALP activation in AVICs. The dye was extracted and quantified as described in the Methods section (*n* = 6, mean ± SD, **P < 0.01 compared with Con; ^#^*P* < 0.05, ^*##*^*P* < 0.01 compared with 7-day culture; ^@^*P* < 0.05, ^@@^*P* < 0.01 compared with 14-day culture).

*In vitro*, the AVICs were calcified over various periods from 7 to 21 days to mimic the process of aged-induced calcification. For further affirming GLP-1 regulated calcification of AVICs with time, we also treated AVICs with 100 pmol/L GLP-1 to identify the time-dependent effect of GLP-1 on calcification from 7 to 21 days. Alizarin Red S staining showed that the extent of calcification rapidly and significantly increased with the time extension (7 days = 129.0 ± 9.2 μg/mg, 14 days = 161.0 ± 6.4 μg/mg, and 21 days = 218.5 ± 7.6 μg/mg compared with 7 days: *P* < 0.01); however, GLP-1 significantly weakened this tendency (respectively, 79.0 ± 7.1, 91.3 ± 10.8, and 108.3 ± 10.5 μg/mg compared with the same groups without GLP-1 treatment: *P* < 0.01) (Figures 4A,B). Compared with the Control groups (7 days = 350.8 ± 28.2 U/mg, 14 days = 495.8 ± 37.2 U/mg, 21 days = 624.8 ± 40.9 U/mg, compared with 7 days: *P* < 0.01), a similar effect of GLP-1 with age (GLP-1: 7 days = 147.2 ± 21.3 U/mg, 14 days = 174.3 ± 19.1 U/mg, and 21 days = 212.8 ± 26.5 U/mg compared with same groups without GLP-1 treatment: *P* < 0.01) was demonstrated in the ALP activation test (Figure 4C).

### GLP-1 Regulated Calcification-Related Gene Expression

**Figure 5:**
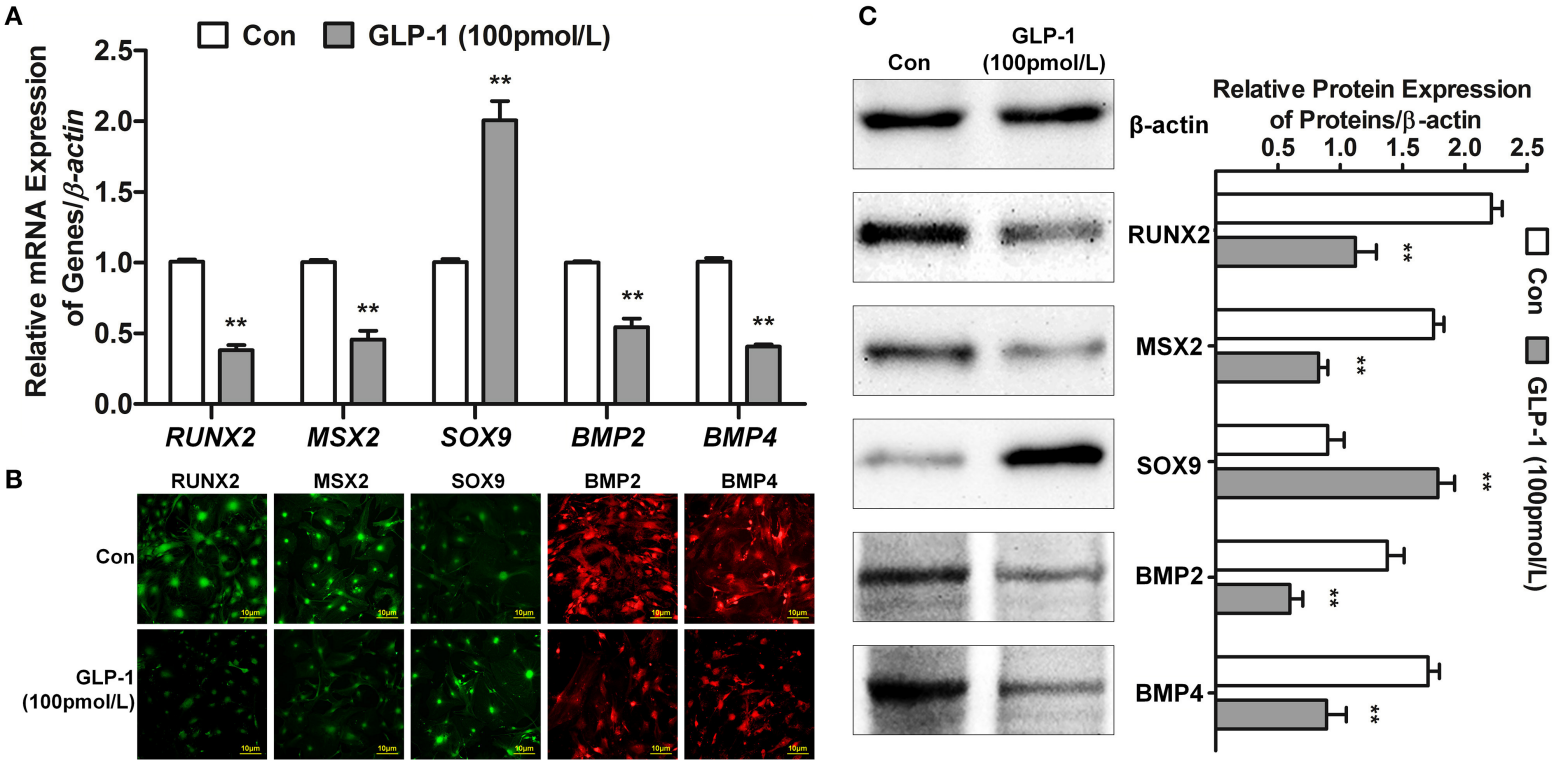
GLP-1 regulates the expression of calcification-related genes. The primary AVICs were stimulated with or without GLP-1 (100 pmol/L) for 21 days in osteogenic medium. The group without the GLP-1 treatment was used as a control (Con). (A) *RUNX2, MSX2, SOX9, BMP2*, and *BMP4* were detected by real-time PCR. **(B)** The protein expression and location of RUNX2, MSX2, SOX9, BMP2, and BMP4 were determined by immunofluorescence analysis. RUNX2, MSX2, and SOX9 were assessed using Alexa Fluor 488-conjugated secondary antibody (Green); BMP2 and BMP4 were assessed using Alexa Fluor 549-conjugated secondary antibody (Red). **(C)** RUNX2, MSX2, SOX9, BMP2, and BMP4 were detected by western blot. The results are representative of three independent experiments. Real-time PCR **(A)** and western blot **(C)** quantified as relative units (genes or proteins/β-actin) (*n* = 3, mean ± SD, **P < 0.01 compared with Con).

Many genes participated in AVIC mineralization, RUNX2 (29), MSX2 (30), and SOX9 (31) act as nuclear transcription factors to regulate downstream gene transcription. For example, the target genes BMP2 and BMP4 (32) promoted calcification of AVICs. Thus, we tested whether GLP-1 inhibited AVIC calcification by regulating the expression of these genes. First, the mRNA levels of *RUNX2, MSX2, SOX9, BMP2*, and *BMP4* were detected by real-time PCR. Glucagon-like peptide-1 decreased the transcription of *RUNX2* by 62% (Control = 1.01 ± 0.02 vs. GLP-1 = 0.38 ± 0.04, *P* < 0.01), *MSX2* by 54% (Control = 1.00 ± 0.02 vs. GLP-1 = 0.46 ± 0.06, *P* < 0.01), *BMP2* by 46% (Control = 1.00 ± 0.01 vs. GLP-1 = 0.54 ± 0.06, *P* < 0.01), and *BMP4* by 59% (Control = 1.01 ± 0.03 vs. GLP-1 = 0.41 ± 0.02, *P* < 0.01) but increased *SOX9* 2.01-fold (Control = 1.00 ± 0.02 vs. GLP-1 = 2.01 ± 0.14, *P* < 0.01) (Figure 5A). Second, the distributions of RUNX2, MSX2, SOX9, BMP2, and BMP4 were identified by immunofluorescence in AVICs. RUNX2, MSX2, and SOX9 were located in the nucleus; BMP2 and BMP4 were expressed throughout the cells. GLP-1 also reduced the levels of RUNX2, MSX2, BMP2, and BMP4 but induced SOX9 (Figure 5B). Finally, the concentrations of these proteins were measured by western blot and analyzed by the IOD value (ratio of proteins/β-actin). GLP-1 decreased the expression of RUNX2 by 49% (Control = 2.21 ± 0.09 vs. GLP-1 = 1.12 ± 0.17, *P* < 0.01), MSX2 by 53% (Control = 1.75 ± 0.08 vs. GLP-1 = 0.83 ± 0.07, *P* < 0.01), BMP2 by 57% (Control = 1.38 ± 0.13 vs. GLP-1 = 0.60 ± 0.10, *P* < 0.01), and BMP4 by 48% (Control = 1.70 ± 0.09 vs. GLP-1 = 0.89 ± 0.16, *P* < 0.01) but increased SOX9 1.98-fold (Control = 0.90 ± 0.13 vs. GLP-1 = 1.78 ± 0.13, *P* < 0.01) (Figure 5C).

### Clinical Characteristics and GLP-1 Level in Serum of Non-CAVD and CAVD Groups

Previous results showed the concentration of GLP-1 decreased in valves of CAVD patients. *In vitro*, GLP-1 reversed mmineralization of AVICs with dose- and time-dependent manner, and GLP-1 downregulated pro-calcification genes expression but upregulated anti-calcification genes expression. The serum of CAVD (*n* = 200) and non-CAVD (*n* = 197) were collected to analysis the relationship of GLP-1 concentration and CAVD.

The demographic and clinical characteristics of the study participants were summarized in Table 2. Compared with the non-CAVD group, the patients in the CAVD group had a higher age range (non-CAVD: 59.02 ± 8.59 years vs. CAVD: 74.14 ± 7.67 years; *P* < 0.001), percentage of hypertension (non-CAVD: 64.5% vs. CAVD: 77.0%; *P* = 0.008), and rate of coronary heart disease (non-CAVD: 68.0% vs. CAVD: 88.5%; *P* < 0.001). There were no significant difference in sex, body mass index (BMI), active smoking, alcohol, percentage of diabetes, fasting glucose, HbA1c or γ-glutamyl transpeptidase between the two groups. The diastolic blood pressure was significantly higher in the CAVD group (Non-CAVD: 131.49 ± 17.73 vs. CAVD: 139.78 ± 20.68; *P* < 0.001), but the systolic blood pressure was significantly lower (Non-CAVD: 77.07 ± 11.70 vs. CAVD: 74.42 ± 11.43; *P* = 0.023). Calcific aortic valve disease patients had higher lipoprotein (A) [Non-CAVD: 0.12 (0.20) vs. CAVD: 0.14 (0.30); P = 0.026] and lower triglycerides [Non-CAVD: 1.78 ± 1.13 vs. CAVD: 1.56 ± 0.86; *P* = 0.025] and LDL (Non-CAVD: 1.13 ± 0.26 vs. CAVD: 1.07 ± 0.29; *P* = 0.044), but not total cholesterol or HDL. Renal and blood urea nitrogen (Non-CAVD: 5.06 ± 1.29 mmol/L vs. CAVD: 6.03 ± 2.40 mmol/L; *P* < 0.001), creatinine (Non-CAVD: 74.07 ± 16.07 mmol/L vs. CAVD: 86.38 ± 24.25 mmol/L; *P* < 0.001), and eGFR (CKD-EPI) (Non-CAVD: 90.39 ± 17.62 ml/min vs. CAVD: 72.29 ± 18.23 ml/min; *P* < 0.001) exhibited significant pathogenic propensity changes. No significant difference was observed in terms of the medication treatment between the Non-CAVD and CAVD groups.

Serum Similar to the IHC result for calcific aortic valves, serum GLP-1 was significantly reduced in the CAVD group [Non-CAVD: median = 14.00 pmol/L (25th−75th percentile 9.34–19.02 pmol/L; range 0.38–39.37 pmol/L) vs. CAVD: median = 11.29 pmol/L (25th−75th percentile 8.17–14.92 pmol/L; range 0.05–31.93 pmol/L), *P* < 0.001] (Table 2).

### GLP-1 as an Independent Factor for CAVD

Based on the multivariable regression analysis, age (OR = 1.255; 95% CI 1.199–1.313), fasting glucose (OR = 0.824; 95% CI, 0.702–0.968), HbA1c (OR = 1.542; 95% CI, 1.171–2.031), HDL (OR = 0.142; 95% CI, 0.045–0.443), BUN (OR = 1.270; 95% CI, 1.032–1.563), and GLP-1 (OR = 0.889; 95% CI, 0.844–0.936) were independently associated with CAVD risk (Model 1 in Table 3). When the GLP-1 quartile (median = 12.31 pmol/L, 25th−75th percentiles 8.68–16.73 pmol/L; range 0.05–39.37 pmol/L) in the Non-CAVD and CAVD groups was included in Model 2 (baseline characteristics of the Non-CAVD and CAVD groups according to GLP-1 level, see Supplementary Table 2), the association between age, fasting glucose, HbA1c, HDL, BUN, and CAVD remained significant. Compared with the lowest quartile, the OR-values of the other quartiles decreased from 0.818 to 0.115. However, only the highest quartile (OR = 0.115; 95% CI, 0.045–0.291) was significantly associated with lower risk of CAVD (Model 2 in Table 3).

**Table 3:** Multivariable regression analysis for the risk of CAVD.

	**Variable**	**B**	**S.E**.	**OR (95% CI)**	***P***
Model 1	Age	0.227	0.023	1.255 (1.199–1.313)	<0.001
	Fasting glucose	−0.193	0.082	0.824 (0.702–0.968)	0.018
	HbA1c	0.433	0.14	1.542 (1.171–2.031)	0.002
	HDL	−1.954	0.582	0.142 (0.045–0.443)	0.001
	BUN	0.239	0.106	1.270 (1.032–1.563)	0.024
	GLP-1	−0.118	0.027	0.889 (0.844–0.936)	<0.001
	Constant	−14.442	1.831		<0.001
Model 2	Age	0.235	0.024	1.265 (1.207–1.325)	<0.001
	Fasting glucose	−0.21	0.083	0.810(0.689–0.953)	0.011
	HbA1c	0.422	0.142	1.525 (1.154–2.016)	0.003
	HDL	−2.027	0.597	0.132 (0.041–0.424)	0.001
	BUN	0.214	0.107	1.239 (1.005–1.527)	0.045
	GLP-1^a^				<0.001
	GLP-1^a^ (8.69–12.31)	−0.201	0.418	0.818 (0.361–1.855)	0.631
	GLP-1^a^ (12.32–16.73)	−0.821	0.436	0.440 (0.187–1.034)	0.06
	GLP-1^a^ (16.74–39.37)	−2.162	0.474	0.115 (0.045–0.291)	<0.001
	Constant	−15.333	1.905		<0.001

*Variable(s) entered in Model 1: Age, Male, Body mass index, Active smoker, Alcohol, Hypertension, Systolic blood pressure, Diastolic blood pressure, Diabetes, Fasting glucose, HbA1c, Coronary heart disease, Triglycerides, Total cholesterol, LDL, HDL, Lipoprotein(a), γ-glutamyl transpeptidase, Blood urea nitrogen, Creatinine, eGFR (CKD-EPI) and GLP-1. GLP-1^a^ was transformed by dividing GLP-1 into quartiles. GLP-1^a^ was a categorical variable in the model, with the lowest quartile (0.050–8.785) as a reference category. Variable(s) entered in Model 2: Age, Male, Body mass index, Active smoker, Alcohol, Hypertension, Systolic blood pressure, Diastolic blood pressure, Diabetes, Fasting glucose, HbA1c, Coronary heart disease, Triglycerides, Total cholesterol, LDL, HDL, Lipoprotein(a), γ-glutamyl transpeptidase, Blood urea nitrogen, Creatinine, eGFR (CKD-EPI), GLP-1^a^. B: unstandardized B coefficient*.

Age represented a remarkable risk factor for CAVD and it presented the largest difference from the Non-CAVD group in this study. Therefore, the interaction between GLP-1 quartile and age as covariates was analyzed by multivariable regression analysis. In this analysis, age, fasting glucose, HbA1c, HDL, and BUN remained independent risk factors associated with CAVD. Compared with the lowest quartile, higher GLP-1 reduced the aged-induced risk of pathogenesis from 0.996 to 0.966 (Table 4). Only the fourth quartile (OR = 0.966; 95% CI, 0.953–0.980) was significantly associated with CAVD (Table 4).

**Table 4:** Multivariable regression analysis for the risk of GLP-1 interacting with Age in CAVD.

**Variable**	**B**	**S.E**.	**OR (95% CI)**	***P***
Age	0.251	0.026	1.285 (1.222–1.351)	<0.001
Fasting glucose	−0.222	0.084	0.801 (0.679–0.944)	0.008
HbA1c	0.437	0.143	1.549 (1.170–2.050)	0.002
HDL	−2.071	0.601	0.126 (0.039–0.409)	0.001
BUN	0.221	0.108	1.247 (1.010–1.540)	0.040
GLP-1^a^ * Age				<0.001
GLP-1^a^ (8.69–12.31) * Age	−0.004	0.007	0.996 (0.983–1.009)	0.550
GLP-1^a^ (12.32–16.73) * Age	−0.013	0.007	0.987 (0.974–1.000)	0.055
GLP-1^a^ (16.74–39.37) * Age	−0.034	0.007	0.966 (0.953–0.980)	<0.001
Intercept	−16.334	1.962		<0.001

GLP-1^a^ was transformed by dividing GLP-1 into quartiles. GLP-1^a^ was a categorical variable in the model, GLP ^a^^*^ age as an interaction term with the lowest quartile (0.05–8.68) by age as a reference category. Variable(s) entered in multivariable regression analysis: age, male, body mass index, active smoker, alcohol, hypertension, systolic blood pressure, diastolic blood pressure, diabetes, fasting glucose, HbA1c, coronary heart disease, triglycerides, total cholesterol, LDL, HDL, Lipoprotein(a), γ-glutamyl transpeptidase, Blood urea nitrogen, creatinine, eGFR (CKD-EPI), GLP-1^a^ by age. B: unstandardized B coefficient.

## Discussion

Although some clinical, genetic, and animal studies have led to a partial understanding of CAVD, truly important advancements in the disease management (such as optimal diagnosis and treatment strategies) remain out of reach. This area of study thus requires further investigations, especially regarding endogenous protective factors. In this study, we found that GLP-1, a negative independent risk factor, was decreased in aortic valve and serum of CAVD patients. Glucagon-like peptide-1 is also associated with reduced the odds of CAVD and inhibited AVIC mineralization by regulating calcification related-genes. Thus, GLP-1 exhibited protective characteristics to antagonize CAVD.

The present study found that age, hypertension, systolic blood pressure, diastolic blood pressure, CAVD, triglycerides, LDL, lipoprotein (A), blood urea nitrogen, creatinine, and eGFR (CKD-EPI) (as pathogenic factors) significantly differed between the Non-CAVD and CAVD groups (Table 2). Among these indicators, age (33), hypertension (4), blood pressure (4), CAD (34), lipoprotein (A) (35), and renal function (36) have been reported to participate in and show pathogenicity associated with CAVD. However, there has been little research regarding the protective factors of CAVD. For these reasons, we focused on GLP-1, which has beneficial effects and is associated with LV diastolic function (37), heart rate (38), cardiac remodeling (39), blood pressure (38), lipid profile (40), and cardiovascular disease independent of adiposity or diabetes (41). Notably, the role of GLP-1 had not been reported in CAVD. We found that the level of GLP-1 was not only decreased in the serum of the CAVD group (Table 2) but was also reduced in calcified aortic valves (Figure 2). Thus, GLP-1 may be associated with CAVD; as expected, multivariable regression analysis found that GLP-1 was a negative independent factor for CAVD (OR = 0.922; 95% CI, 0.887–0.958) (Table 3) and significantly weakened the odds risk of CAVD. These results indicate that the variation in GLP-1 concentration affects CAVD. To determine the influence of GLP-1 concentration on CAVD, GLP-1 concentration was divided into quartiles. The patients with the highest quartile of GLP-1 showed the lowest rate of CAVD (31.3%, *P* < 0.001; Supplementary Table 2), and the highest quartile showed a significantly strong negative correlation with CAVD risk (OR = 0.115; 95% CI, 0.045–0.291), which demonstrated that a high dose of GLP-1 exerted an antagonistic effect on the odds risk of CAVD (Table 3). Thus, GLP-1 is a novel protective factor negatively associated with CAVD, and decreases in GLP-1 lead to the progressive calcification of the aortic valve.

Glucagon-like peptide-1 is an incretin hormone that is secreted into the serum by enteroendocrine L-cells (distal ileum and colon) and K-cells (duodenum and jejunum) (42); however, we found GLP-1 in non-mineralized aortic valve regions with or without calcified lesions, which indicates that GLP-1 was secreted from intestinal cells and recruited to the aortic valve to influence the function of AVICs. Glucagon-like peptide-1 localizes to interstitial spaces and tissues to regulate metabolic diseases, such as diabetes and obesity (43, 44); GLP-1 also regulates cell functions to protect against cardiovascular disease. *In vitro* and *in vivo* atherosclerosis studies demonstrate that GLP-1 promotes vasodilatation and suppresses the inflammatory response in endothelial cells, inhibits lipid uptake and inflammatory activity in macrophages, and represses the proliferation of smooth muscle cell (SMCs) to prevent atherosclerosis progression (45). In arterial calcification, similar to bone formation, VSMCs differentiate to the osteoblastic phenotype to play a key role in arterial calcification; however, GLP-1 inhibits osteoblastic differentiation and calcification in human VSMCs (20). There are several similarities between CAVD and arterial calcification; however, AVIC heterogenization and mineralization are key components of the cytopathology in CAVD, and these components differ from those in arterial calcification. In this study, the level of GLP-1 decreased by 19.3% in CAVD serum (Table 2) and 39% in calcified aortic valve (Figure 2), which indicated that a reduction in GLP-1 in the aortic valve caused AVIC calcification. However, whether GLP-1 can reverse CAVD by regulating AVIC osteoblastic differentiation and calcification was not known. To investigate this, we added various doses of GLP-1 to AVICs during the standard process of calcification. Glucagon-like peptide-1 significantly attenuated the density of Alizarin Red S and the activation of ALP at higher doses (Figure 3), which demonstrated that GLP-1 could attenuate CAVD by preventing the mineralization of AVICs. Considering this plus the results of Table 3, we hypothesized that high GLP-1 weakens the risk of CAVD. Our results indicate that GLP-1 inhibits the calcification of AVICs to exert its protective function in CAVD.

Calcific aortic valve disease is a chronic degenerative disease that has multiple risk factors, including diabetes (46), hypertension (4), dyslipidemia (36), and kidney disease (47). The Multi-Ethnic Study of Atherosclerosis (MESA) found that non-Hispanic whites had the highest frequency of CAVD, followed by Hispanics and blacks, which indicates that CAVD presents racial differences (48). Our study in a Chinese population found that age, fasting glucose, HbA1c, HDL, BUN, and GLP-1 were independent risk factors for CAVD, which indicates that age, diabetes, dyslipidemia and renal insufficiency were associated with CAVD in a Chinese population.

Although multiple pathogenic factors take part in CAVD, age is an important and irreversible risk factor and has the strongest correlation with CAVD (48). A previous study showed that more than 50% of patients with aortic valve calcification were older than 75 years, whereas severe stenosis was found in 2–3% of this elderly population (49). And some reports shown with aged, AVICs can form calcium node (50). In our experiments, we found in extended-duration *in vitro* calcification culture, the mineralization level of AVICs increased (Figure 4). These results illustrate that age plays a key role in CAVD mineralization. Moreover, we previously found an effect of GLP-1 on neuroprotection via its reversal of age-induced neurodegeneration, such as that in Alzheimer's and Parkinson's diseases (51).We observed that GLP-1 not only inhibited mineralization but also reduced time-dependent calcification in AVICs from 7 to 21 days (Figure 4). These results demonstrate that the leading risk factor of CAVD may can be attenuated by GLP-1.

RUNX2, MSX2, SOX9, BMP2, and BMP4 are important proteins related to calcification. RUNX2 is an osteogenic and chondrogenic transcription factor that is regulated in multiple manners (52). RUNX2 is upregulated in atherosclerotic calcification and endochondral mineralization programs (29). Hydrogen peroxide activates osteogenic Cbfa1/RUNX2 (53) and MSX2/Wnt signaling (30), thereby enhancing mineralization. Miller et al. also found that both of these regulatory cascades were activated in calcifying human aortic valves (54). Acharya et al. (31) demonstrated that Notch1 maintains SOX9 expression to inhibit osteogenic mineralization in AVICs. BMP2 and BMP4 increase the secretion of OPN by upregulating ALP, resulting in the degradation of tissue pyrophosphate (32). Glucagon-like peptide-1 attenuates osteoblastic differentiation and calcification by inhibiting ALP, osteocalcin (OC), and RUNX2 in human VSMCs (20), but whether GLP-1 regulates the expression of MSX2, SOX9, BMP2, and BMP4 remains unknown. As we observed in this study, GLP-1 decreased the expression of RUNX2, MSX2, BMP2, and BMP4 but increased the expression of SOX9 in AVICs (Figure 5), which were first suggested a relationship between GLP-1 and these genes in the AVIC calcification process. These results indicate that GLP-1 reversed mineralization in AVICs via two pathways, first by inhibiting the expression of osteogenic genes and second by promoting the expression of anti-osteogenic genes. These results indicate that GLP-1 reversed mineralization in AVICs via two pathways, first by inhibiting the expression of osteogenic genes and second by promoting the expression of anti-osteogenic genes.

This study showed that the level of GLP-1 decreased in both the local calcific aortic valve and in the serum of CAVD patients and that this decrease was associated with age. This indicated that GLP-1 could have value in predicting the occurrence and development of CAVD.

## Study Limitations

In this study, we examined the role of GLP-1 in CAVD; however, some *in vivo* experiments and details of the molecular mechanism were lacking. We found HDL and fasting glucose to be negative independent risk factors associated with CAVD. HDL has anti-oxidative and anti-inflammatory properties, but the role of HDL in CAVD is not clear. Interestingly, our study found that fasting glucose was associated with a reduced risk of CAVD and that high glucose reversed AVIC calcification (data not shown); however, the mechanisms of these effects remain unknown. HDL and fasting glucose in CAVD will be investigated in further studies. Moreover, as a single center cross-sectional study with retrospective characteristic, it might be susceptible to center biases or recall bias. Also, as a retrospective study, the study only indicated associations but not formulate causal relationships. Although we took multiple clinical important cofounders into consideration, it might be possible that unknown potential factors may be missed and it might interfere in our findings. Finally, CAVD was only assessed by echocardiography. Cardiac computed tomography is also another useful approach to quantify aortic valve calcium burden. Finally, although we carefully controlled for the major known confounders, unknown factors may still have interfered in our findings.

Therefore, the results remained to be further confirmed in larger sample size study with prospective randomized controlled designs.

## Conclusion

Valve tissue and serum from CAVD patients were characterized with lower level of GLP-1. Clinical and cellular evidence suggests that GLP-1 participates in the pathological calcification of the aortic valve. Calcific aortic valve disease is highly prevalent in the elderly, and there are currently no absolute effective treatments to reverse its progression. This study reveals some novel characteristics of GLP-1 and its potential therapeutic value for CAVD.

## Data Availability Statement

The raw data supporting the conclusions of this article will be made available by the authors, without undue reservation.

## Ethics Statement

The studies involving human participants were reviewed and approved by Shanghai Ninth People's Hospital+Shanghai Jiaotong University School of Medicine. The patients/participants provided their written informed consent to participate in this study. The animal study was reviewed and approved by Shanghai Ninth People's Hospital+Shanghai Jiaotong University School of Medicine.

## Author Contributions

YL and KY designed the study, performed data analysis and interpretation, and drafted the manuscript. FX, QZha, and QZhang performed data collection and analysis. FX, QW, and YY contributed to interpretation, drafting, and editing the manuscript. YL and KY provided study design, interpretation, wrote and edited manuscript, are the guarantors of this work and takes full responsibility for the work as a whole, including the study design, access to data, and the decision to submit and publish the manuscript. All authors contributed to conception and design, acquisition of data or analysis and interpretation of data, drafting the article or revising it critically for important intellectual content, and gave final approval of the version to be published.

## Conflict of Interest

The authors declare that the research was conducted in the absence of any commercial or financial relationships that could be construed as a potential conflict of interest.

## Publisher's Note

All claims expressed in this article are solely those of the authors and do not necessarily represent those of their affiliated organizations, or those of the publisher, the editors and the reviewers. Any product that may be evaluated in this article, or claim that may be made by its manufacturer, is not guaranteed or endorsed by the publisher.
