# Supplementary material for: Decreased Glucagon-Like Peptide-1 Is Associated With Calcific Aortic Valve Disease: GLP-1 Suppresses the Calcification of Aortic Valve Interstitial Cells
Source: Front Cardiovasc Med. 2021 Aug 26;8:709741. doi: 10.3389/fcvm.2021.709741 (PMC8428521; doi:10.3389/fcvm.2021.709741)
Supplement: Supplementary file 1 [file Table_1.docx]

**Supplementary Table 1.** The primer has been used for Realtime-PCR.

| Gene | Forward primer | Reverse primer | products size (bp) |
| --- | --- | --- | --- |
| *RUNX2* | 5'-CTCTACTATGGCACTTCGTC-3' | 5'-CTTCCATCAGCGTCAACAC-3' | 164 |
| *MSX2* | 5'-CTCCAGCCTGCCCTTCA-3' | 5'-CACGCCGCTCCATCTTC-3' | 219 |
| *SOX9* | 5'-AGGTGCTCAAAGGCTACGACTG-3' | 5'-CGCGGCTGGTACTTGTAATCC-3' | 295 |
| *BMP2* | 5'-TGACGAGGTCCTGAGCG-3' | 5'-CCTGAGTGCCTGCGATA-3' | 126 |
| *BMP4* | 5'-AGCATCCCTGAGAACGAG-3' | 5'-CGAGTCTGATGGAGGTGAG-3' | 299 |
| *β-actin* | 5'-CGTGGACATCCGCAAAG-3' | 5'-TGGAAGGTGGACAGCGA-3' | 201 |
