# Supplementary material for: Decreased Glucagon-Like Peptide-1 Is Associated With Calcific Aortic Valve Disease: GLP-1 Suppresses the Calcification of Aortic Valve Interstitial Cells
Source: Front Cardiovasc Med. 2021 Aug 26;8:709741. doi: 10.3389/fcvm.2021.709741 (PMC8428521; doi:10.3389/fcvm.2021.709741)
Supplement: Supplementary file 2 [file Table_2.docx]

**Supplementary Table 2. Baseline characteristics of study population according to GLP-1 level.**

|  | GLP-1 concentration, pmol/L | | | |  |
| --- | --- | --- | --- | --- | --- |
|  | **First quartile (0.05-8.68)** | **Second quartile (8.69-12.31)** | **Third quartile (12.32-16.73)** | **Fourth quartile (16.74-39.37)** | ***P*** |
| n | 99 | 100 | 99 | 99 |  |
| CAVD, n (%) | 57(57.6%) | 61(61.0%) | 51(51.5%) | 31(31.3%) | <0.001 |
| Age, yrs | 66.2±10.67 | 68.11±10.85 | 67.41±11.39 | 64.77±11.38 | NS |
| Male, n (%) | 59(59.6%) | 59(59.0%) | 57(57.6%) | 59(59.6%) | NS |
| Body mass index, kg/m^2^ | 24.67±3.74 | 24.77±2.99 | 24.95±3.46 | 25.17±3.52 | NS |
| Active smokers, n (%) | 24(24.2%) | 24(24.0%) | 26(26.3%) | 33(33.3%) | NS |
| Alcohol, n (%) | 9(9.1%) | 11(11.0%) | 11(11.1%) | 14(14.1%) | NS |
| Hypertension, n (%) | 66(66.7%) | 70(70.0%) | 71(71.7%) | 74(74.7%) | NS |
| Diabetes, n (%) | 31(31.3%) | 22(22.0%) | 26(26.3%) | 45(45.5%) | 0.002 |
| Coronary heart disease, n (%) | 76(76.8%) | 79(79.0%) | 78(78.8%) | 78(78.8%) | NS |
| Systolic blood pressure, mmHg | 76.01±11.29 | 73.75±11.67 | 75.27±10.90 | 77.93±12.38 | NS |
| Diastolic blood pressure, mmHg | 137.00±20.84 | 133.66±18.69 | 136.68±19.35 | 135.35±19.95 | NS |
| Fasting glucose, mmol/L | 5.41±1.56 | 5.67±2.84 | 5.42±1.83 | 5.51±1.68 | NS |
| HbA1c, % | 6.19±1.03 | 6.31±1.31 | 6.26±1.22 | 6.42±1.22 | NS |
| Triglycerides, mmol/L | 1.47±.75 | 1.67±1.08 | 1.62±0.75 | 1.93±1.30 | 0.012 |
| Total cholesterol, mmol/L | 3.90±1.03 | 4.07±1.24 | 4.13±1.18 | 3.89±1.05 | NS |
| LDL, mmol/L | 1.13±0.31 | 1.09±0.26 | 1.12±0.30 | 1.06±0.22 | NS |
| HDL, mmol/L | 2.33±0.88 | 2.39±0.98 | 2.50±1.00 | 2.19±0.84 | NS |
| Lipoprotein(a), g/L | 0.13(0.19) | 0.12(0.24) | 0.16(0.28) | 0.12(0.26) | NS |
| γ-glutamyl transpeptidase, U/L | 21.00(17.00) | 21.00(15.50) | 19.00(16.00) | 19.00(22.00) | NS |
| Blood urea nitrogen, mmol/L | 5.44±1.54 | 5.60±1.82 | 5.67±2.06 | 5.49±2.45 | NS |
| Creatinine, mmol/L | 81.89±21.55 | 80.33±22.76 | 78.74±19.80 | 80.12±21.89 | NS |
| eGFR(CKD-EPI), mL/min | 79.82±17.49 | 79.63±19.02 | 82.77±21.60 | 82.88±21.90 | NS |
| Metformin, n (%) | 19(19.2%) | 12(12.0%) | 12(12.1%) | 20(20.2%) | NS |
| Statin, n (%) | 87(87.9%) | 81(81.0%) | 81(81.8%) | 81(81.8%) | NS |

Values are expressed as the mean ± SD, number (%), or median (interquartile range). Linear regressions were used to evaluate relations of GLP-1 to baseline characteristics and calculate P values. CAVD: calcific aortic valve disease; HDL: high-density lipoprotein; LDL: low-density lipoprotein; NS: not significant.
